# Postoperative Analgesic Efficacy of Thoracic Paravertebral Block and Erector Spinae Plane Block Combination in Video-Assisted Thoracic Surgery

**DOI:** 10.7759/cureus.15614

**Published:** 2021-06-12

**Authors:** Musa Zengin, Ramazan Baldemir, Gulay Ulger, Hilal Sazak, Ali Alagoz

**Affiliations:** 1 Anesthesiology and Reanimation, University of Health Sciences, Ankara Atatürk Chest Diseases and Thoracic Surgery Training and Research Hospital, Ankara, TUR

**Keywords:** paravertebral block, erector spinae plane block, postoperative analgesia, video-assisted thoracic surgery, multimodal analgesia

## Abstract

Background

The combination of a thoracic paravertebral block (TPVB) and erector spinae plane block (ESPB) has not been investigated. We aimed to evaluate the effects of the combination of TPVB and ESPB particularly on postoperative pain scores in patients undergoing video-assisted thoracic surgery (VATS).

Methods

From January 1, 2021, to March 1, 2021, 13 patients older than 18 years who underwent combined ESPB and TPVB for analgesic treatment after elective VATS were included in the study. Standard anesthesia induction was performed for all patients, and the block was performed in the lateral decubitis position before surgery. Using the in-plane technique, an ultrasound (US)-compatible 22-gauge, 8-mm nerve block needle was introduced 2-3 cm lateral to the spinous process of the T6 vertebra and advanced in the caudocranial direction. Fifteen (15) ml of 0.25% bupivacaine was administered and pleural depression was observed. The same needle was withdrawn from the paravertebral space and advanced into the interfascial plane above the transverse process and below the erector spinae muscle at the T5 level. Then, 15 ml of 0.25% bupivacaine was injected.

Results

The combination of TPVB and ESPB was performed in 13 patients. The mean age was 44.3 (21-68) years. The mean body mass index (BMI) was 23.21 (16.9-35.9) kg/m^2^. Postoperative 24 hours morphine consumption was 24.5 (16-42) mg. In three cases, visual analog scale (VAS) scores at rest were ≥4; therefore, tramadol (25 mg, IV) was given as an additional analgesic. Nausea and vomiting were observed in only one case in the early postoperative period.

Conclusıons

As a new technique, the combination of TPVB and ESPB in this preliminary study provided effective postoperative pain management along with the use of morphine in acceptable quantities. Large-scale, randomized-controlled, and comparative studies are needed to demonstrate the efficacy of the combination of TPVB and ESPB.

## Introduction

Video-assisted thoracic surgery (VATS) has become a popular thoracic surgical technique in recent years. VATS is a less invasive and less painful procedure as compared to thoracotomy [1]. Thoracic epidural analgesia (TEA) remains to be the gold standard in the treatment of postoperative pain in thoracic surgery [2-4]. Thoracic paravertebral block (TPVB) is another technique for pain management after VATS [5]. Serratus anterior plane block (SAPB) is a new technique for providing surgical anesthesia and postoperative analgesia [5]. Ultrasound-guided (US-guided) ESBP is an interfascial plane block described by Forero et al. [6] for the treatment of thoracic neuropathic pain [7]. Interfascial plane blocks reduce opioid consumption without motor blocks, such as neuraxial blocks, providing adequate long-lasting postoperative analgesia [8-9]. However; the occurrence of side effects, such as hypotension, nausea, and urinary retention after TEA, limits its use in patients undergoing VATS [10]. Such undesirable effects are less common in the TPVB and ESPB procedures [1,10-12]. The number of case reports and the number of prospective randomized controlled studies about US-guided ESBP are increasing in the literature. In general, comparative studies on TPVB and ESPB are conducted and their analgesic effects are evaluated in studies [12-13]. It is known that the use of analgesic drugs and techniques in combination yields a multimodal effect leading to the increased analgesic efficiency, especially in patients with intense postoperative pain such as patients undergoing thoracic surgery [1,14]. Consensus has not been reached yet about the spread of local anesthetics in anatomical spaces and the volume to be administered in regional analgesia, which is part of multimodal regimens in the treatment of pain after thoracic surgery [13,15]. Therefore, considering that the analgesic effect would possibly be increased by combining TPVB and the newly introduced technique, ESPB, we aimed to evaluate the effects of the combination of TPVB and ESPB particularly on postoperative pain scores in patients undergoing VATS.

## Materials and methods

During the pre-anesthesia evaluation, 13 patients who were scheduled for VATS under general anesthesia were informed about TPVB and ESPB. Then, informed consent was obtained from the patients. Premedication with midazolam was administered to the patients, who were monitored in the operating room in accordance with the American Society of Anesthesiologists (ASA) standards. Following preoxygenation, anesthesia was induced with 2 mg/kg propofol, 1.5 mcg/kg fentanyl, and 0.1 mg/kg vecuronium. After the intubation with a left-sided double-lumen endobronchial tube, anesthesia was maintained by administering sevoflurane in an oxygen and air mixture and by administering remifentanil infusion at a dose of 0.01-0.1 mcg/kg/min. Before the commencement of the surgical procedure, a combination of TPVB and ESPB was performed under ultrasonography guidance.

Technique

**Figure 1:**
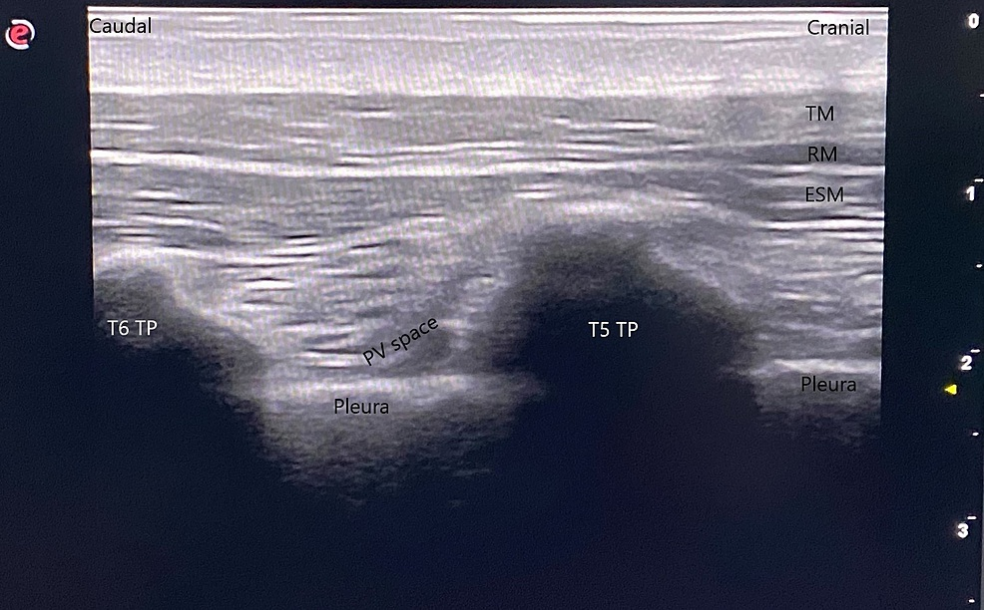
Anatomical scene before the block The transverse process, muscles reaching the transverse process (the trapezius, rhomboid major, and erector spinae muscles), and the paravertebral space were scanned. (ESM: Erector spinae muscles, PV space: Paravertebral space, RM: Rhomboid major, TM: Trapezius muscle, TP: Transverse process)

The block procedure was performed under general anesthesia before the skin incision in order to prevent the patient's anxiety and ensure comfort. Thus, a preemptive effect was also achieved. Following the anesthesia induction, the TPVB and ESBP combination was performed under US guidance when the patients were in the lateral decubitus position. After skin antisepsis, the needle insertion area was covered with sterile drapes. The high-frequency 6-18 MHz linear probe (MyLab Six, Esaote, Genoa, Italy) in a sterile cover was placed 2-3 cm lateral to the spinous process of the T5 vertebra. The transverse process, muscles reaching the transverse process (the trapezius, rhomboid major, and erector spinae muscles), and the paravertebral space were scanned (Figure 1).

**Figure 2:**
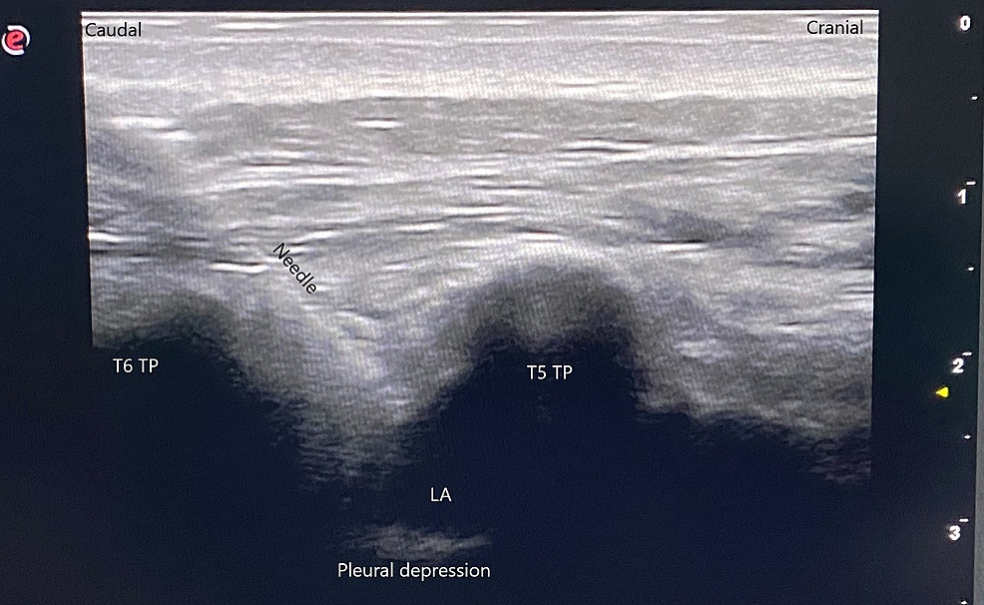
Paravertebral block and pleural depression Fifteen ml of 0.25% bupivacaine was administered and pleural depression was observed. (LA: Local anesthetic, TP: Transverse process)

Then, using the in-plane technique, a US-compatible 22-Gauge, 8-mm nerve block needle (Pajunk, SonoPlexSTIM, Germany) was introduced 2-3 cm lateral to the spinous process of the T6 vertebra and advanced in the caudocranial direction. The needle was advanced until it reached the paravertebral space at the T5 level. Then, 15 ml of 0.25% bupivacaine was administered and pleural depression was observed (Figure 2).

**Figure 3:**
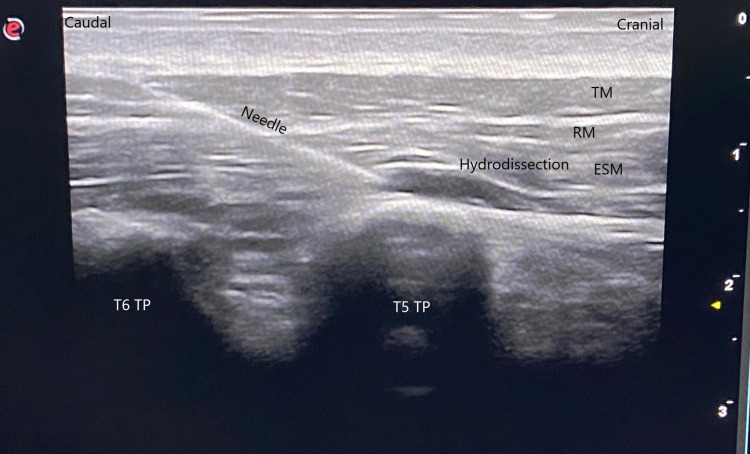
Hydrodissection Hydrodissection with 2 ml of saline solution was performed into the interfascial plane above the transverse process and below the erector spinae muscle. (ESM: Erector spinae muscles, RM: Rhomboid major, TM: Trapezius muscle, TP: Transverse process)

The same needle was withdrawn from the paravertebral space and advanced into the interfascial plane above the transverse process and below the erector spinae muscle at the T5 level. Hydrodissection with 2 ml of saline solution was performed in this area, observing that the needle was in the correct location (Figure 3).

**Figure 4:**
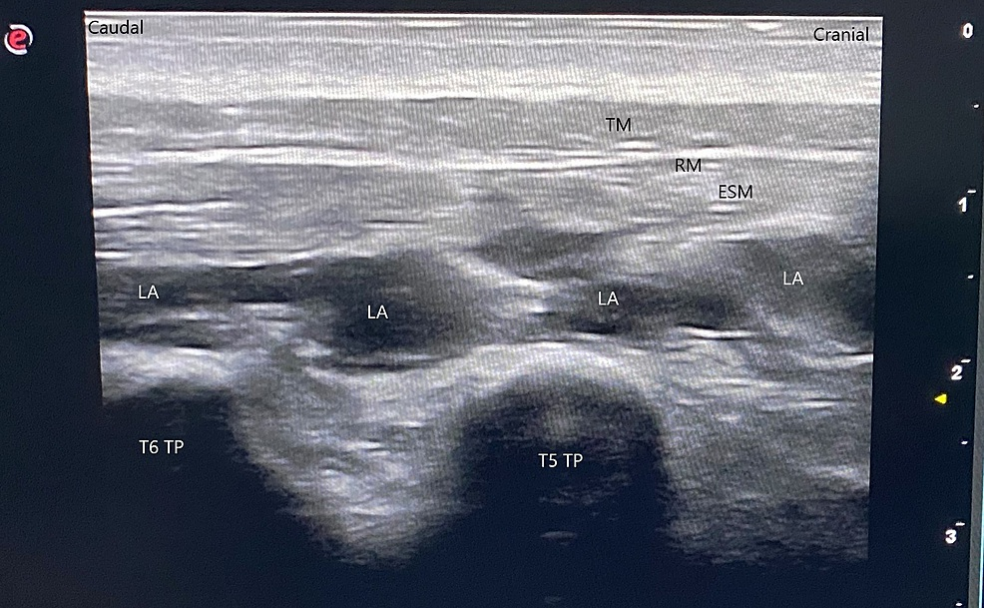
Erector spinae plane block Figure 4. The local anesthetic spread caudally and cranially beneath the erector spinae muscle (ESM: Erector spinae muscles, LA: Local anesthetic, RM: Rhomboid major, TM: Trapezius muscle, TP: Transverse process).

Then, 15 ml of 0.25% bupivacaine was injected. During the injection, it was observed that the local anesthetic spread caudally and cranially beneath the erector spinae muscle (Figure 4).

At the end of the surgical procedure, tramadol at a dose of 1.5 mg/kg and 50 mg dexketoprofen were administered via the intravenous route slowly. Following extubation, the patients were transferred to the postoperative intensive care unit.

Postoperative analgesia

In the postoperative surgical intensive care unit, intravenous morphine was administered via patient-controlled analgesia (PCA) pump for 24 hours. Pain intensity was evaluated using the 10-point visual analog scale (VAS). The PCA pump’s dose delivery was limited to administer a bolus dose of 1 mg morphine and deliver a maximum dose of 12 mg morphine in total within four hours with lockout intervals of 15 minutes. Paracetamol 1 g was administered intravenously every eight hours for multimodal analgesia. A rescue analgesic agent, 25 mg tramadol was given to patients intravenously when a score of VAS at rest ≥ 4. Patients who were transferred to the ward at the postoperative 24^th^ hour were given tramadol 50 mg capsule every eight hours and 500 mg paracetamol tablet and dexketoprofen 50 mg tablet every 12 hours. Static and dynamic VAS scores were recorded at the postoperative 30^th^ minute, first hour, sixth hour, 12^th^ hour, and 24^th^ hour. The need for additional analgesics and side effects, such as allergic reactions, respiratory depression, sedation, urinary retention, nausea-vomiting, and itching, were recorded.

## Results

The combination of TPVB and ESPB was performed in 13 patients. There was a male predominance in the patient group (92.3%). The mean age was 44.3 (21-68) years. The mean body mass index (BMI) was 23.21 (16.9-35.9) kg/m^2^. The shortest and the longest operation times were 80 and 150 minutes, respectively. The shortest and longest anesthesia durations were 105 and 175 minutes, respectively. The mean dose of remifentanil needed intraoperatively was 468.84 (201-757) mcg. All patients underwent VATS and wedge resection (Table 1).

**Table 1:** Demographic and surgical characteristics of cases ASA: American Society of Anesthesiologists, BMI: Body mass index, F: Female, M: Male

Case Number	Age (year)	Gender	ASA Score	BMI kg/m^2^	Duration of Surgery / Anesthesia (min)	Total remifentanil requirement (mcg)
1	50	M	2	19.0	80 / 105	372
2	60	M	2	35.9	150 / 175	757
3	40	M	2	27.7	120 / 145	552
4	46	M	2	20.4	150 / 175	510
5	21	M	1	16.9	110 / 135	330
6	65	M	3	20.4	80 / 110	201
7	40	M	2	19.6	120 / 135	383
8	30	M	2	17.5	150 / 170	478
9	44	M	2	22.6	100 / 125	502
10	40	M	2	27.2	90 / 115	372
11	51	F	2	29.5	130 / 155	512
12	21	M	1	18.4	150 / 175	554
13	68	M	2	26.7	130 / 160	572
Mean	44.3			23.21	120.0 / 144.61	468.84

The mean dose of morphine needed postoperatively was 24.5 (16-42) mg. Postoperative VAS scores were presented in Table 2.

**Table 2:** Postoperative pain scores and morphine consumption VAS: Visual analog scale

Case Number	VAS Scores (Static/Dynamic)					Postoperative Morphine Consumption (mg)
	30 min	1 h	6 h	12 h	24 h	
1	4 / 5	3 / 4	2 / 3	2 / 3	1 / 2	28
2	1 / 2	2 / 3	1 / 2	1 / 2	1 / 1	18
3	3 / 4	3 / 4	2 / 3	2 / 3	2 / 3	18
4	1 / 2	1 / 2	0 / 1	0 / 1	0 / 1	22
5	2 / 3	1 / 2	1 / 2	1 / 2	1 / 2	16
6	2 / 3	2 / 3	2 / 3	1 / 2	1 / 2	24
7	4 / 5	3 / 4	2 / 4	2 / 3	1 / 2	42
8	1 / 2	1 / 2	1 / 2	0 / 1	0 / 1	19
9	2 / 4	2 / 4	2 / 3	2 / 3	1 / 3	22
10	4 / 5	3 / 4	2 / 4	2 / 3	2 / 3	36
11	3 / 4	3 / 4	2 / 3	1 / 2	1 / 2	24
12	1 / 2	1 / 2	0 / 2	0 / 1	0 / 1	22
13	3 / 4	3 / 4	2 / 3	2 / 3	1 / 2	28
Mean	2.3 / 3.4	2.1 / 3.2	1.4 / 2.6	1.2 / 2.2	0.9 / 1.9	24.5 (16-42)

In three cases, VAS scores at rest were ≥4; therefore, tramadol (25 mg, IV) was given as an additional analgesic. Nausea was observed in only one case (Case 13) in the early postoperative period.

## Discussion

The results of this study have shown that compared to the literature, the combination of TPVB and ESPB enabled the maintenance of lower VAS scores in the early postoperative period in patients, who underwent wedge resection through VATS under general anesthesia. In addition, the need for additional analgesics was limited and side effects were observed in only one patient.

TEA is considered the gold standard technique in pain management after thoracic surgery [2-4,10,14]. However, the use of anticoagulants, the invasive nature of the procedure, and difficulties associated with needle insertion limit the use of TEA. Moreover, complications such as sympathetic blockade, respiratory depression, and urinary retention are not uncommon [14]. Outcomes of TEA and TPVB use for analgesia after VATS are still controversial [11,16-17].

In recent years, ESPB has become a widely used method to provide postoperative analgesia after VATS. However, the results reported by studies comparing the analgesic efficacy of TPVB, intercostal block, and ESPB are contradictory [18-20]. In a study conducted by Çiftçi et al. [21], it was observed that a single dose of ESPB provided effective postoperative analgesia compared to the control group. In addition, the authors reported that fewer side effects occurred in the ESPB group compared to the control group. In our study, although there was not a control group, we observed that pain scores and the need for additional analgesics were limited in the postoperative 24 hours. Furthermore, only nausea and vomiting were observed in a patient.

TPVB is used in various clinical situations, including thoracic surgery, rib fractures, and chronic neuropathic pain [10,11,14]. Although it has recently been suggested that ESPB can be a favorable option because it has been shown in studies that it can provide analgesia at equivalent or close levels compared to TPVB [18-19,21]; Chen et al. observed that morphine consumption was higher in ESPB compared to multiple-injection TPVB and intercostal blocks [20]. A study conducted by Zhang et al. [22] on healthy volunteers reported that when ESPB provided a widespread block in the posterior wall of the thorax, only the dorsal nerves were blocked. These results show that the spread of the local anesthetic in ESPB and the analgesic efficacy of ESPB has not been elucidated completely. Furthermore, there is no consensus regarding the volume of local anesthetics to be administered. In a cadaveric study, Diwan et al. have postulated that the effect of ESPB occurs via the spread in epidural and paravertebral spaces [13]. It was observed in another cadaveric study that as the volume of the anesthetic increased in ESPB, the spread to the posterior muscles and fascial layers increased predominantly compared to the extent of the paravertebral spread [23].

These results show that no consensus has been achieved yet regarding the efficacy of ESBP, the volume to be administered to achieve the appropriate blockade and the spread of the local anesthetic in ESBP; even when ESBP is performed under US guidance. However, consistent with the mechanism of action of multimodal analgesia, we think that the combination of TPVB and ESPB may not only provide a synergistic blockade effect but also compensate potential failures associated with either of these two blockade techniques. In our study, we used 15 ml of 0.25% bupivacaine to perform both blockade techniques. Considering its spread, we think that this volume may be efficacious in a wide anatomical region.

In our study, TPVB and ESPB were performed in the same session and via a single needle insertion. Although there are studies; in which ESPB is applied in the caudocranial direction, the technique is frequently applied in the craniocaudal direction and at the level of T5 [18,21-22]. In our study, for ease of application, we advanced the needle in the caudocranial direction so that the paravertebral space would be better localized. Besides, ESPB was applied after TPVB in this study. We think that observing the spread of the local anesthetic during both procedures may be guiding to confirm the success of the blockade.

This study has some limitations. First, TPVB and ESPB were performed under general anesthesia, therefore, the assessment of the dermatomal levels after blockades could not be performed. However, the nerve blocks were performed under ultrasound guidance and the entire spread of local anesthetics was observed during injections. Second, this study is a preliminary case-control study without a control group. Comprehensive, randomized controlled studies could provide precise results about the efficacy of the combination of TPVB and ESPB.

## Conclusions

In conclusion, the combination of TPVB and ESPB in this preliminary study enabled effective postoperative pain management along with the use of morphine in acceptable quantities. Another advantage of this technique is that it provides a safer and more comfortable block for the patients and practitioners due to the simultaneous use of two different blocks with the insertion of a single needle. At the same time, a more effective multimodal analgesia can be provided by combining intravenous analgesia with two different block techniques. Furthermore, the limited postoperative complications due to analgesia may be related to the combination of these two blocks. Large-scale, comprehensive, prospective, and randomized-controlled studies are needed to demonstrate the efficacy of the combination of TPVB and ESPB.
